# Psychological Wellbeing and Life Satisfaction among Chinese Older Immigrants in Canada across the Early and Late Stages of the COVID-19 Pandemic

**DOI:** 10.3390/healthcare12181899

**Published:** 2024-09-22

**Authors:** Lixia Yang, Andrea D. Y. Lee, Linying Dong

**Affiliations:** 1 Department of Psychology, Toronto Metropolitan University, Toronto, ON M5B 2K3, Canada; dya.lee@torontomu.ca; 2 Ted Rogers School of Information Technology Management, Toronto Metropolitan University, Toronto, ON M5G 2C3, Canada; ldong@torontomu.ca

**Keywords:** wellbeing, life satisfaction, social support, Chinese older adults, COVID-19

## Abstract

Objectives: This study compared the psychological wellbeing, life satisfaction, and perceived social support in Chinese older immigrants living in Canada between the early (i.e., Wave 1: September–November 2020) and late (i.e., Wave 2: January–February 2023) stages of the COVID-19 pandemic. Additionally, it assessed perceived social support from family, friends, or others as predictors for psychological wellbeing and life satisfaction in this population. Methods: These questions were addressed with a cross-sectional survey design with two independent samples at Wave 1 (n = 171) and Wave 2 (n = 191), respectively. Results: The results revealed lower levels of psychological wellbeing, life satisfaction, and perceived social support in Wave 2 compared to Wave 1. The hierarchical regression models identified social support from friends (but not from family or others) as a significant predictor for psychological wellbeing and life satisfaction. Additionally, a higher level of income and being a male predicted better psychological wellbeing. A higher level of income and a lower level of education predicted greater life satisfaction. Conclusions: The findings suggest a deterioration in psychological wellbeing and life satisfaction from the early to late stages of the pandemic and highlight the protective effect of social support from friends among Chinese older immigrants.

## 1. Introduction

As of May 2023, the World Health Organization (WHO) no longer classified the pandemic as a global emergency [1]. However, the COVID-19 pandemic and the related regulation measures (e.g., lockdown, social distancing, and cessation of community activities) have had long-lasting, adverse psychological outcomes such as increased depression and anxiety [2]. Longitudinal studies have shown that anxiety, depression, and COVID-19-related stress increased during the COVID-19 pandemic [3]. A systematic review of 294 articles across the globe reported a general mental health deterioration during the first wave of the COVID-19 pandemic, supporting the concept of the “psychological COVID-19 syndrome” [4].

### 1.1. Psychological Impacts of the Pandemic on Older Adults

Older adults are at a higher risk of contracting COVID-19 and more likely to develop severe symptoms and complications [5]. Higher COVID-related mortality rates have been reported for older adults compared to younger adults, especially for those with comorbid conditions such as hypertension, cardiovascular disease, and diabetes [6]. Additionally, older adults reported an increased risk of frailty, anxiety, depression, loneliness, cognitive decline, worsened mental health, and lower life satisfaction during the pandemic [7]. Reduced social support, lack of community belonging, and barriers to using online technology or the internet to stay socially connected have negatively affected older adults’ psychological wellbeing during the pandemic [5,8,9]. In Canada, a study found that Chinese immigrant older (>64 years old) and middle-aged adults (25–64 years old) reported higher psychological distress compared to younger adults [10]. Nevertheless, results are still mixed on the impacts of the pandemic on older adults’ psychological wellbeing and life satisfaction.

#### Psychological Wellbeing and Life Satisfaction during the Pandemic

Psychological wellbeing can be defined as overall positive views and feelings towards the various life domains [11]. Life satisfaction refers to the evaluation of one’s life circumstances and the assessment of the feelings and attitudes about one’s life [12]. These two concepts describe emotional and cognitive aspects of one’s life, respectively.

It has been reported that psychological wellbeing declined following the onset of the pandemic, but the results are mixed on the psychological wellbeing fluctuation over the course of the pandemic. For example, a longitudinal study in Denmark revealed decreased mental health in 2020 compared to 2019, with a slight increase in 2021 compared to 2020 [13]. Another study conducted in Canada reported that levels of psychological wellbeing did not change significantly over the course of the pandemic [14]. Relatedly, past studies on life satisfaction revealed a similar pattern, with a general decline during compared to before the pandemic [15]. In Canada, this decline was steeper in immigrants from Asia relative to immigrants from Europe and Australia or individuals from the US [15]. In a study conducted in Australia, Wright and colleagues [16] found that life satisfaction decreased between the first and second waves of the pandemic, and lower social connectedness was significantly associated with lower life satisfaction. On the other hand, other studies have found that life satisfaction stayed stable in older adults during the pandemic [17].

In light of the mixed results, the current study aims to assess and compare the psychological wellbeing and life-satisfaction of Chinese older immigrants in Canada between the early (2020) and late (2023) stages of the pandemic and identify associated predictors, such as perceived social support from different sources.

### 1.2. Perceived Social Support during the Pandemic

Perceived social support is the subjective measure of anticipated or expected social aid [18]. According to the stress buffering hypothesis proposed by Cohen and Wills [19], high levels of perceived social support can be protective against the adverse effects of stress. In line with this hypothesis, previous research has found that perceived social support serves as an external resource to buffer the social isolation and loneliness resulting from COVID-19 and the related lockdown measures [20]. A study by Li and colleagues [21] found that social support and resilience were protective factors of mental health during the pandemic. It was reported that social support was protective against loneliness, depression, nervousness, and sleep problems among older adults during the pandemic [22]. Xu and colleagues [23] found that social support moderated the relationship between loneliness and anxiety across three timepoints (i.e., before, the peak, and the decline stages of the pandemic). Despite its importance in mental health, perceived social support has been reported to decrease during the pandemic, presumably due to the social distancing and lockdown measures [21].

Nevertheless, previous research suggested that people have turned to alternative methods of social interaction, such as social contact through digital technologies [24]. More older adults used the internet during compared to before the pandemic, and this contributed to the level of their social contact and interactions with family members, which alleviated loneliness [20]. As a result, the general level of social support or their social link with close family remained unchanged during the pandemic [23]. However, the impacts of older adults’ online social networking varied, with bonding (forming relationship with family and friends) mitigating whereas bridging (forming broader casual social networks) heightened COVID-19-related anxiety [8]. Furthermore, the impacts of online social networking were also moderated by negative information dissemination [8].

#### Social Support from Different Sources

Although high levels of social support from family, friends, communities, organizations, and society (as a whole) were in general associated with better mental health and acted as a buffer against low levels of resilience during the pandemic [21], the benefits might vary according to the sources/types of social support for individuals from different cultural backgrounds. In line with the family-oriented collectivism culture in Chinese populations [25], studies have found that social support from family was associated with lower levels of depression and loneliness and higher levels of self-esteem and life satisfaction compared to social support from friends among Chinese older adults [26,27].

It should be noted, however, that Chinese older immigrants reported having limited sources of support [28,29]. Nevertheless, it has been reported that they rely on different sources for different types of support. For example, they rely on their adult children primarily for personal, instrumental, or financial support, but rely on friends, neighbors, and religious community primarily for general information, advice, companionship and emotional support [28,29]. A study by Tsai and Lopez [30] found that Chinese older immigrants identified family support as most satisfying compared to support from other sources. On the other hand, it has been found that having more friends, beyond close family relationships, was significantly associated with lower levels of depression in Chinese older adults [31]. Based on the literature, both family and friend support may be important to Chinese older immigrants. This study aimed to address the relative importance of social support from friends, family, and others in Chinese older immigrants’ psychological wellbeing and life satisfaction at the early and late stage of the pandemic.

### 1.3. Chinese Immigrants and COVID-19 Pandemic

Past research found that immigrant older adults were more likely to experience loneliness than those who were born in Canada, and immigrants who migrated as adults were at an even higher risk of loneliness [32]. Beyond the impacts of COVID-19 on physical and mental health, Chinese oversea residents have experienced unique challenges including increased anti-Asian racism and disapproval of political criticism targeting China [33]. Studies have reported detrimental psychological impacts of the racial discrimination against Chinese, both perceived and experienced, among Chinese immigrants in Canada during the COVID-19 pandemic [34]. Furthermore, the collectivism of Chinese culture may promote an extended family structure [25] and thus make social support from the family especially important. However, Chinese older adults in Canada had lowered expectations of filial piety from children [35] and this may decrease the relative importance of family/children support in their psychological wellbeing. In light of these earlier works, it is important to assess the relative importance of social support from difference sources in Chinese older immigrants’ psychological wellbeing and life satisfaction, particularly at the start and the end stage of the pandemic, as they were associated with drastically different social support dynamics.

In this context, the current study aimed to address the following three questions: (1) do Chinese older immigrants in Canada report different levels of psychological wellbeing, life satisfaction, and perceived social support between the early (September–November 2020) and late (January–February 2023) stage of the pandemic? (2) does perceived social support (from family, friends, and others) predict psychological wellbeing and life satisfaction of this population across the two time points? (3) are there any persistent sociodemographic predictors for psychological wellbeing and life satisfaction of this population across the two time points? Based on the previous literature on COVID-19 and Chinese older adults [30,31], we hypothesized that all sources of social support would be positively associated with psychological wellbeing and life satisfaction.

## 2. Materials and Methods

### 2.1. Participants

Participants for both Wave 1 and Wave 2 surveys were recruited through widely and publicly distributed registration posts, social media platforms (e.g., WeChat), the internet, websites, or community email lists. The participants were self-screened based on the following inclusion criteria: (1) aged 65 or over; (2) Chinese migrants (e.g., Canadian citizens, immigrants, visitors); (3) have lived or plan to live in Canada for at least 6 months; and (4) able to read and write in Mandarin. Both surveys targeted Chinese older immigrants. The Wave 1 survey was initially attempted by 208 individuals, with 171 eligible and completed respondents (i.e., completed more than 85% of survey items) included in the final sample (age *M* = 74.23, *SD* = 5.69, 109 women, completion rate = 82.21%). The Wave 2 survey was initially attempted by 273 participants, with 191 eligible and completed respondents (i.e., completed more than 85% of survey items) included in the final sample (age *M* = 75.19, *SD* = 5.98, 136 women, completion rate = 69.96%).

Wave 1 survey data were collected in September to November 2020 when Canada was in the second wave of the pandemic with an increase in new cases and tightened public health restrictions. Wave 2 survey data were collected in January to February 2023, a subsequent period of the pandemic, when most restrictions were lifted [36]. The two surveys were completed by two independently recruited samples, without tracking for repeated participation. Table 1 presents the sample characteristics.

### 2.2. Measures

All the measures in the two surveys were translated from English into simplified Chinese by bilingual researchers on the team and verified through back-translation. Discrepancies were resolved by group discussion under the supervision of the project lead. The surveys were piloted by some Chinese older adults to ensure the wording was culturally relevant and understandable before being distributed. The surveys were built in Qualtrics^TM^. The Wave 1 survey was part of a larger project [9], and the Wave 2 survey was based on the Wave 1 survey. Both surveys included the following overlapped questions and measures: (1) sociodemographic predictive variables (i.e., age, sex, marital status, education, employment status, family income, resident status, birthplace, length in Canada, housing style, housing size, and religion); (2) outcome measures, including the 5-item WHO Well-being Index (WHO-5) [37] and the Satisfaction with Life Scale (SWLS) [12]; (3) the primary predictor measure: the Multidimensional Scale of Perceived Social Support (MSPSS) [38].

#### 2.2.1. Outcome Measures

The World Health Organization-five wellbeing index. The WHO-5 [37] measures the level of overall psychological wellbeing during the past two weeks. It includes five items (e.g., “I have felt cheerful in good spirits”) based on a 6-point Likert scale ranging from 0 (at no time) to 5 (all the time). Following the scoring instructions of the WHO-5 [39], the sum score was multiplied by 4 to get a total score ranging from 0 to 100, with higher scores indicating better psychological wellbeing. The internal reliability at Wave 1 and Wave 2 were α = 0.89 and α = 0.94, respectively.

Satisfaction with life scale. The SWLS [12] assesses global cognitive judgment of life satisfaction with five items (e.g., “I am satisfied with my life”) based on a 7-point Likert scale ranging from 1 (strongly disagree) to 7 (strongly agree). The sum score ranged from 5 to 35, with higher scores indicating higher satisfaction. The internal reliability at Wave 1 and Wave 2 were α = 0.85 and α = 0.90, respectively.

#### 2.2.2. Primary Predictor Measure

Multidimensional scale of perceived social support scale. The MSPSS [38] assesses perceived social support from others, family, and friends with 12 items (4 items for each sub-scale) based on a 7-point Likert scale ranging from 1 (very strongly disagree) to 7 (very strongly agree). For example, “There is a special person who is around when I am in need” describes support from others; “My family really tries to help me” assesses support from family; and “My friends really try to help me” depicts support from friends. Considering that different social support groups (from community, family, and friends) offer different types of support [28,29] and demonstrate different psychological benefits in Chinese older adults [30,31], the three subscales were scored independently and used as three predictive variables in the final analysis. The sum score for each sub-scale ranged from 4 to 28, with higher scores indicating higher perceived support. The internal reliability at Wave 1 and Wave 2 were α = 0.92 and α = 0.96, respectively.

### 2.3. Procedure

The survey at the two waves received approval from the affiliated University Research Ethics Board [REB2020-247, REB2022-393]. The two surveys were conducted independently without directly tracking repeated participation. For both waves, participants could complete the survey on their own by clicking the survey link or scanning the survey QR code; or register to participate with a research assistant over the phone or Zoom (individually or in small groups). Although more participants completed the survey with assistance at Wave 1 than Wave 2, unfortunately, this completion mode information was not collected or recorded to enable further analysis of its effect on the results.

Prior to participating, all participants were provided a brief description of the study with a link to the full consent form. Informed consent was collected by clicking “yes” to the question “do you agree to participate in this survey?” before the participants could officially start the survey. For those who had individual testing sessions, a research assistant facilitated the survey completion or completed the survey on behalf of the participant based on their spoken responses. In group Zoom sessions, the survey was completed by the participants through a distributed survey link posted in the Zoom chat. Throughout the session, participants could ask questions and seek support in a private breakout room with a research assistant. Participants were clearly informed in the consent form that if they chose to complete the survey through a Zoom or phone meeting, their participation would not be entirely confidential. However, all personal contact information was kept confidential and only used in scheduling or sending survey-related information. Survey data were coded by randomly assigned subject ID, and participants’ identity information was never linked to their responses. Upon the completion of the Wave 1 or Wave 2 surveys, they received a small token or a chance to participate in a prize draw for digital gift cards as compensation for their time. The compensation was open to all participants regardless of whether they completed one or both surveys. In any event, the incentive amount and method may not affect the results given the previous finding of little effect of an incentive on survey response accuracy [40].

### 2.4. Data Analysis Approach

Data analysis was conducted in IBM SPSS 28.0. For sample characteristics, Chi square tests were conducted to compare the categorical sociodemographic variables (see Table 1), and *t*-tests were conducted to compare age, social support, life satisfaction, and psychological wellbeing (see Table 2) between Wave 1 and Wave 2 samples. Participants with missing data were minimal (n ≤ 6) across the critical measures (predictive or outcome variables) in the two data sets. Those who missed more than half of the items for a specific measure would be excluded from the analysis involving that specific measure. For those who missed half or fewer items for a specific measure, the missing data points were replaced by the average of that scale or subscale for each participant. Univariate ANOVA models were conducted on each of the two outcome variables (psychological wellbeing and life satisfaction) to identify significant sociodemographic predictors following a conventional cut-off criterion of *p* ≤ 0.20 [41]. Pearson correlations were conducted to assess the association between primary predictors (social support general and subscale scores) and outcome variables. Subsequently, two 2-step hierarchical linear regression models were conducted, one for psychological wellbeing and the other for life satisfaction. In Step 1, the data collection Wave (1 vs. 2) and perceived social support from others, family, and friends were entered as the primary predictors. In Step 2, the sociodemographic covariates identified by the univariate ANOVA models were added. A listwise deletion approach was used to remove missing data points. All beta values were reported as absolute values in the Section 3 to enable comparability.

## 3. Results

### 3.1. Sample Characteristics and Wave Differences

Chi square tests (see Table 1) were used to compare the categorical sociodemographic variables between Wave 1 and Wave 2. The two samples were largely comparable in sociodemographic profile (*p*s ≥ 0.052) except for housing type (*X*^2^ = 24.43, *p* < 0.001), where more people in Wave 2 (23%) reported “other” option than in Wave 1 (5%). However, participants in different house types did not significantly differ in the two outcome variables, WHO-5 and SWLS (*p*s ≥ 0.477, Table 3), and therefore house type was not considered further in the subsequent regression models. Additionally, the two samples also did not differ in average age (*t* = −1.51, *p* = 0.132, Table 2).

**Table 3:** Sociodemographic Group Differences in WHO-5 and SWLS Outcome Scores.

Variables		WHO-5 Mean (SD)	*F*	*p*	SWLS Mean (SD)	*F*	*p*
Wave	1 = 2020	17.62 (4.79)	47.87 ***	<0.001	26.19 (4.57)	57.02 ***	<0.001
	2 = 2023	13.13 (5.68)			21.64 (5.12)		
Sociodemographic Variables							
Sex	Female	14.59 (5.83)	**2.60**	0.108	23.46 (5.60)	0.09	0.771
	Male	16.33 (5.31)			24.29 (4.67)		
Marital status	Married/Partnered	15.32 (5.61)	0.01	0.920	23.87 (5.35)	0.48	0.489
	Other	15.01 (6.06)			23.58 (5.44)		
Education	≤High school	15.40 (5.34)	0.07	0.789	25.20 (4.72)	8.46 **	0.004
	≥College/University	15.17 (5.88)			23.28 (5.50)		
Employment status	Retired	15.14 (5.76)	0.05	0.820	23.75 (5.39)	0.10	0.757
	Other	16.28 (5.35)			24.25 (5.12)		
Family income	Low	14.80 (5.63)	8.45 **	0.004	23.27 (5.47)	16.90 ***	<0.001
	Moderate/High	16.40 (5.88)			25.19 (4.82)		
Resident status	Citizen/Permanent resident	15.22 (5.78)	0.004	0.951	23.71 (5.38)	0.12	0.726
	Other	15.57 (4.73)			25.53 (4.88)		
Birthplace	Mainland China	15.10 (5.74)	**2.66**	0.104	23.68 (5.40)	4.21 *	0.041
	Other	17.89 (5.04)			25.83 (4.23)		
Length in Canada	0–5 yrs	14.85 (5.86)	0.67	0.513	23.86 (5.43)	**1.90**	0.151
	6–15 yrs	15.55 (5.77)			24.23 (4.99)		
	>15 yrs	14.94 (5.64)			23.00 (5.86)		
Housing type	Apartment	16.07 (5.18)	0.51	0.601	24.64 (5.11)	0.74	0.477
	House	15.13 (6.10)			23.86 (5.37)		
	Other	13.48 (5.51)			21.46 (5.39)		
Housing Size	1 person	14.85 (6.16)	0.56	0.570	23.80 (6.11)	1.25	0.288
	2 persons	15.60 (5.71)			24.00 (5.08)		
	3 persons or more	14.81 (5.58)			23.44 (5.50)		
Religion	No	15.38 (5.74)	0.79	0.340	23.83 (5.25)	0.14	0.710
	YES/Other	14.88 (5.72)			23.70 (5.66)		

Note. *F* and *p* values refer to the Univariate ANOVA results. Bolded *F* values (*p* < 0.20) refer to the potential predictors to be entered as sociodemographic covariates in the regression model displayed in Table 4. * *p* < 0.05, ** *p* < 0.01, *** *p* < 0.001.

**Table 4:** Linear Regression Models for WHO-5 and SWLS.

Step	Predictors		WHO-5				SWLS			
			β	95% CI	*F*	*R*^2^	β	95% CI	*F*	*R*^2^
1	Wave	1 = 2020 (Ref.)			45.65 ***	0.35			44.82 ***	0.36
		2 = 2023	−3.04 ***	(−4.07, −2.01)			−3.22 ***	(−4.18, −2.27)		
	MSPSS (Other)		−0.04	(−0.85, 0.76)			0.17	(−0.59, 0.92)		
	MSPSS (Family)		0.76	(−0.10, 1.61)			0.66	(−0.14, 1.46)		
	MSPSS (Friends)		2.02 ***	(1.36, 2.68)			1.67 ***	(1.06, 2.29)		
2	Wave	1 = 2020 (Ref.)			28.93 ***	0.38			24.67 ***	0.39
		2 = 2023	−3.03 ***	(−4.04, −2.02)			−3.15 ***	(−4.09, −2.21)		
	MSPSS (Other)		−0.26	(−1.06, 0.55)			−0.03	(−0.77, 0.72)		
	MSPSS (Family)		0.68	(−0.17, 1.52)			0.64	(−0.15, 1.43)		
	MSPSS (Friends)		2.14 ***	(1.49, 2.79)			1.75 ***	(1.14, 2.36)		
	Sex	Female (Ref.)								
		Male	1.29 *	(0.23, 2.35)			X	X		
	Education	≤High school (Ref.)								
		≥College/University	X	X			−1.17 *	(−2.21, −0.14)		
	Family income	Low (Ref.)								
		Moderate/High	1.34 *	(0.24, 2.43)			1.66 **	(0.63, 2.69)		
	Birthplace	Mainland China (Ref.)								
		Other	1.87	(−0.49, 4.23)			1.38	(−0.67, 3.44)		
	Length in Canada	0–5 yrs (Ref.)								
		6–15 yrs	X	X			1.08	(−0.17, 2.32)		
		>15 yrs	X	X			0.30	(−1.06, 1.65)		

Note. Ref. = reference. *β* refers to unstandardized *β*. CI = Confidence Interval. * *p* < 0.05, ** *p* < 0.01, *** *p* < 0.001.

The wave differences in continuous predictive and outcome variables were assessed with a set of independent two-sample *t*-tests. The results showed significantly lower levels of perceived social support (overall and from each source: others/family/friends), psychological wellbeing, and life satisfaction, in Wave 2 relative to Wave 1 samples (*p*s < 0.001, Table 2).

### 3.2. Potential Predictors of Outcome Variables

A univariate ANOVA was conducted to assess categorical sociodemographic group differences in the two outcome variables and thus identify potential sociodemographic predictors based on the cut-off score of *p* ≤ 0.020 (Table 3) [41]. For psychological wellbeing (i.e., WHO-5), the following potential predictors were identified: sex (female vs. male), family income (low vs. moderate/high), and birthplace (Mainland China vs. other), *p*s ≤ 0.108. For life satisfaction (i.e., SWLS), the following potential predictors were identified: education (≤high school vs. ≥ college/university), family income, birthplace, and length in Canada (0–5 years, 6–15 years, and >15 years), *p*s ≤ 0.151.

According to the Pearson correlation analyses (Table 2), social support (general score and the three subscale scores) showed moderately to strong positive correlations with psychological wellbeing as indexed by WHO-5 (*r*s ≥ 0.43, *p*s ≤ 0.001) and life satisfaction as indexed by SWLS (*r*s ≥ 0.42, *p*s ≤ 0.001).

### 3.3. Regression on Psychological Wellbeing

Table 4 displays the results from the 2-step hierarchical linear regression on psychological wellbeing (i.e., WHO-5). Both Step 1 (Wave and social support subscales as predictors) and Step 2 (sociodemographic covariate added, including sex, family income, and birthplace) explained a significant portion of the variance in WHO-5 (Step 1: *R*^2^ = 0.35, *F* = 45.65, *p* < 0.001; Step 2: *R*^2^ = 0.38, *F* = 28.93, *p* < 0.001).

Both testing waves and social support from friends were identified as significant predictors for psychological wellbeing before (*βs* ≥ 2.02, *p*s ≤ 0.001) and after controlling for sociodemographic covariates (*β*s ≥ 2.14, *p*s ≤ 0.001). After controlling for sociodemographic covariates, the Wave 2 sample scored 3.03-unit lower on psychological wellbeing (i.e., WHO-5) than the Wave 1 sample. Additionally, for every 1-unit increase in social support from friends (i.e., the MSPSS-friends score), there was an increase of 2.14-unit in the psychological wellbeing (i.e., the WHO-5 score). However, social support from others or family were not significant predictors for psychological wellbeing (*β*s ≤ 0.68, *p*s ≥ 0.118). Furthermore, sex and family income were identified as significant sociodemographic predictors for psychological wellbeing (*β*s ≥ 1.29, *p*s ≤ 0.018). Males and those with moderate/high levels of family income reported a higher level of psychological wellbeing compared to females and those with low family income, respectively.

### 3.4. Regression on Life Satisfaction

Based on the 2-step hierarchical linear regression model on life satisfaction (Table 4), both Step 1 (wave and social support subscales as predictors) and Step 2 (sociodemographic covariate added, including education, family income, birthplace, and length in Canada) explained a significant portion of the variance in SWLS (Step 1: *R*^2^ = 0.36, *F* = 44.82, *p* < 0.001; Step 2: *R*^2^ = 0.39, *F* = 24.67, *p* < 0.001).

Both testing waves and social support from friends were identified as significant predictors for life satisfaction before (*β*s ≥ 1.67, *p*s ≤ 0.001) and after controlling for sociodemographic covariates (*β*s ≥ 1.75, *p*s ≤ 0.001). After controlling for sociodemographic covariates, the Wave 2 sample scored 3.15-unit lower on life satisfaction (i.e., SWLS) than the Wave 1 sample. Also, for every 1-unit increase in social support from friends (i.e., the MSPSS-friends score), there was an increase of 1.75-unit in life satisfaction (i.e., the SWLS score). However, social support from others or family were not significant predictors for life satisfaction (*β*s ≤ 0.64, *p*s ≥ 0.112). Furthermore, education and family income were identified as significant sociodemographic predictors for life satisfaction (*β*s ≥ 1.17, *p*s ≤ 0.027). Those with a college/university or higher education and those with a low family income reported a lower level of life satisfaction than those with a high school or lower education and those with a moderate/high family income, respectively.

## 4. Discussion

Taken together, the current study revealed the following main findings: (1) perceived social support, psychological wellbeing, and life satisfaction were reported to be significantly lower at the late (2023) compared to the early (2020) stage of the pandemic; (2) perceived social support from friends, but not family or others, was positively associated with psychological wellbeing and life satisfaction across the two testing waves; (3) higher family income is protective of psychological wellbeing and life satisfaction, males reported better psychological wellbeing than females, and lower education was associated with better life satisfaction.

### 4.1. Wave Differences: The Early vs. Late Stage of the Pandemic

The decline in social support, psychological wellbeing, and life satisfaction during the pandemic was largely consistent with previous studies that found a decrease in mental health [3], wellbeing [13,14], life satisfaction [15,16], and social support [20,22] after and during the pandemic. The current study extended these results to the end stage of the pandemic and to Chinese older adults living in Canada.

However, it should be noted that the results are inconsistent with other studies that found a stable level of wellbeing in Canada [14] and an even slight increase in life satisfaction in Finnish older adults [17]. One possible explanation for the discrepancies is that these earlier studies were conducted at the earlier time points (in 2020 or 2021) when individuals were probably adapting or accepting to live their lives with the pandemic. A longitudinal study in Denmark revealed a U-shaped pattern, with mental health declining at the beginning of the pandemic in 2020 compared to 2019, combined with a slight improvement by 2021 compared to 2020 [13]. The data in Canada followed a similar U-shaped pattern. Specifically, the proportion of participants reporting high levels of life satisfaction reduced during February to May 2021 compared to September to December of 2020 [41]. But the proportion of participants who reported high levels of life satisfaction was similar or slightly higher in February to May of 2023 compared to 2020 [42]. Unfortunately, our study did not have data collected in 2021 or 2022; thus, we would not be able to depict the trajectory in mental health status across the full pandemic spectrum.

Additionally, these earlier studies were conducted largely with non-immigrant populations. It is possible that immigrant older adults may face extra barriers (e.g., language, cultural, service and support, and discrimination and racism) and thus demonstrate continuously worsened psychological wellbeing and life satisfaction up to the end of the pandemic. This aligns well with the findings of an earlier study [15] of a steeper decline in life satisfaction among Asian immigrants than others in Canada. Taken together, these results alarmingly suggest that the pandemic has had a prolonged detrimental impact on Chinese older immigrants’ psychological wellbeing and life satisfaction.

### 4.2. The Prediction of Social Support for Wellbeing and Life Satisfaction

In support of the hypothesis, the results indicated social support, from friends specifically, as a significant protective predictor for both psychological wellbeing and life satisfaction, even after controlling for testing wave and sociodemographic covariates. This is largely in line with previous findings of a positive impact of social support on wellbeing and mental health during the pandemic (e.g., [9,20,21,22]). It supports the relationship between lower social connectedness and lower life satisfaction [16].

Particularly, friends’ support has been identified as a critical factor for older adults’ emotional health. For example, German older adults reported that activities with friends were associated with better life satisfaction, increased positive affect, and decreased negative affect, but activities with family members were associated with increased positive or negative affect but not with life satisfaction [43]. In another study, older adults reported that encounters with friends were more pleasant and that they had fewer discussions of stressful experiences compared to encounters with family members [44]. In Chinese older adult immigrants, friends’ support had a greater influence on positive affect compared to family or spouse support, whereas children’s support had a greater impact on reduced negative affect [45]. Similarly, studies have found that older Chinese immigrants with many friends also had lower levels of depressive symptoms [46]. In sum, the findings highlight the importance of social support and interactions with friends in Chinese older adults’ psychological wellbeing.

Previous research has revealed that social support from the family is an important protective factor of psychological wellbeing and life satisfaction in Chinese older adults [26,27]. Considering the collectivistic culture that promotes an extended family structure [25], we had hypothesized that social support from the family would be associated with better psychological wellbeing and life satisfaction. However, the results indicated that social support from family and others were not significant predictors for psychological wellbeing or life satisfaction (see Table 4), despite the significant positive correlations between the two (see Table 2). One possible reason for this finding may be because Chinese older immigrants had lowered expectations of family support, especially from children. For example, Zhang [35] found that Chinese older adults in Canada had lowered expectations of filial piety from their children, possibly due to concerns about their children’s minority status in Canada. Additionally, Chinese older immigrants reported concerns regarding becoming a burden to their children [30,47]. Therefore, the concerns and pressures of adapting to a new country may weaken the relationship between family social support and psychological wellbeing or life satisfaction.

Furthermore, family relationships can also be a source of stress among immigrants. In a systematic review of Chinese and Korean older immigrants, Guo and Stensland [48] found that negative family and social interactions seemed to be strongly associated with depressive symptoms. Negative intergenerational relationships and spousal criticism were found to be related to higher depressive symptoms in older Chinese immigrants as well [46]. In sum, positive family interactions may be beneficial but negative family interactions are detrimental to psychological wellbeing comparatively.

### 4.3. Sociodemographic Predictors for Psychological Wellbeing and Life Satisfaction

The results of this study also identified being a male as a positive predictor for better psychological wellbeing whereas having lower education was a positive predictor for better life satisfaction, and higher family income was protective of both psychological wellbeing and life satisfaction (See Table 4).

The finding that gender is a significant predictor of psychological wellbeing is in line with previous research findings that women experienced higher levels of distress, depression, anxiety, stress, and problems with sleep compared to men during the COVID-19 quarantine [49]. Researchers suggested that this may be in part due to gender-specific coping strategies or expectations [49]. For example, previous studies have found that female students showed a higher level of social support seeking coping and emotional coping whereas males showed higher levels of meaning-focused coping and problem solving [50,51]. Therefore, the COVID-19 pandemic-related social restrictions may have a greater negative impact on female participants’ wellbeing compared to male participants. Another study conducted in India found that fear of COVID-19 was positively related to perceived stress and negatively related to wellbeing and life satisfaction, and this relationship was stronger among women compared to men [52]. Additionally, a study found that in China, female participants reported higher levels of psychological stress compared to male participants, and the researchers suggested that this may be related to females’ increased care burden at home and being more heavily impacted by COVID-19 at work [53].

The protective effect of a lower education (high school and under) on life satisfaction is somewhat counterintuitive and inconsistent with some previous work which suggests a positive relationship between higher education and better life satisfaction in Chinese and Korean older immigrants [54] and a lower level of resilience in less-educated individuals during the pandemic [4]. Nevertheless, it supports some other studies with Chinese participants [53,55]. One possible explanation for this apparently counterintuitive finding was that higher levels of education are usually associated with higher demands or life expectations, which might diminish their life satisfaction [55]. This explanation is supported by the paradox of choice [55], namely the more options one has, the less satisfied one would feel about the decision [56]. Therefore, the larger number of choices/opportunities associated with higher levels of education may lead to lower life satisfaction. Furthermore, those with a higher education might be more likely to hold a stable job before the pandemic and thus their daily routine might be more likely to be disrupted by the pandemic, which may lead to lower life satisfaction. Therefore, the cognitive dissonance, or the discomfort caused by conflicting thoughts [57], between the expectation of better employment and financial stability from higher levels of education and the reality of job instability and financial burden due to the pandemic may explain their lower life satisfaction. However, further research is needed to validate these speculations underlying the relationship between education and life satisfaction.

The positive impact of family income levels on psychological wellbeing and life satisfaction somewhat supports previous findings that a lower income was detrimental to mental health during the pandemic (see a review by Filindassi and colleagues [4]). Among Chinese immigrants in Canada, it was also found that financial status was negatively associated with psychological distress and loneliness and positively associated with mental health status [10,34]. In a study of Chinese and Korean older immigrants, having a higher income was associated with higher life satisfaction [54]. The economic consequences of the pandemic have had negative impacts on mental health, as demonstrated in previous research that financial stress during the pandemic was significantly associated with decreased mental health [58].

### 4.4. Limitations and Future Directions

This study has several limitations. First, its cross-sectional study design does not allow us to draw causal conclusions from the findings. Second, the convenient sampling procedure may limit the representativeness of the sample. Additionally, without a comparison between immigrant and non-immigrant older adults, it is unclear whether the reported results could be generalized to the general population in Canada. The results might be restricted to Chinese older adults currently residing in Canada, active on WeChat, email communication, and/or internet, and fluent in Mandarin. Third, the results might be affected by a desirability bias given the self-reported nature of a survey study. Fourth, only the most likely significant predictors were entered in the final regression models. This approach may not capture other factors that might be important but were not captured by the survey or the ANOVA. Fifth, the study compared data collected from two independent samples, and thus the results might be affected by group differences that have not been accounted for in this study. Sixth, no data were collected in 2021–2022, which limited our ability to track the results pattern throughout different stages of the pandemic. Lastly, the response mode might play a role considering that more participants responded to the survey with assistance through Zoom/phone calls in the Wave 1 than in the Wave 2 data collection. Unfortunately, the response mode was not recorded; thus, there is no way to assess its impacts on the results. Nevertheless, past research verified the feasibility of on-line survey data collection with older adults [59], and it was reported that the survey response accuracy/honesty did not vary according to the privacy level, ranging from potentially identifiable to completely anonymous, of the survey administration [40]. Furthermore, relative to web-based survey respondents, paper respondents were more likely to report higher depression symptoms and a lower health level [60]. Somewhat inconsistently, our study revealed a poorer psychological wellbeing and life satisfaction at Wave 2 (more web-based respondents) than Wave 1 (more RA-assistant respondents). This suggests that the results of the current study are unlikely attributed to the response mode differences between the two waves.

Future research is needed. For example, longitudinal studies would be more effective in revealing the causal relationship between social support, psychological wellbeing, and life satisfaction. Additionally, qualitative studies would allow us to further delve into the specific importance of social support from friends for the wellbeing among Chinese older immigrants in Canada. In addition, future work might further examine the relative importance across the different sources of social support (family, friends, others) in older adults’ psychological wellbeing and life satisfaction to pinpoint the specific theoretical or practical foundations of these effects.

## 5. Conclusions

Nevertheless, it should be noted that this study made some important novel contributions to the literature. First, the results extended the previous findings of the detrimental psychological impacts of the pandemic to the end stage of the pandemic and a vulnerable population, Chinese older adults in Canada. Second, the results provided insights into changes of these impacts from the initial early to the later end stage of the pandemic. Importantly, psychological wellbeing, life satisfaction, and social support were reported to be lower at the late stage (2023) relative to the early stage (2020) of the pandemic, probably due to the lingering effect of the prolonged pandemic. Lastly, the results innovatively highlight perceived social support from friends as an important protective factor for psychological wellbeing and life satisfaction among Chinese older immigrants in Canada. It is possible that the pandemic may continue to have lingering and long-term mental health consequences, thus it is important to provide continuous and sustainable mental health services to best support a vulnerable minority population [61]. The findings have implications for social and health services and programs to promote social support and thus enhance the psychological wellbeing and life satisfaction of Chinese older immigrants. The results highlight that having close friends is especially important. Based on this result, community programs for this population could be specifically tailored to heavily focus on friendship-forming and socialization.

## Figures and Tables

**Table 1:** Sample Categorical Sociodemographic Characteristics across the Two Waves of Data Collection.

Categorical Variables		Wave 1: 2020 (*n*/%)	Wave 2: 2023 (*n*/%)	*Χ* ^2^	*p* (2-Sided)
Sex	Female	109 (63.7)	136 (71.2)	1.23	0.292
	Male	53 (31.0)	51 (26.7)		
Marital status	Married/Partnered	121 (70.8)	136 (71.2)	0.01	1.00
	Other	50 (29.2)	55 (28.8)		
Education	≤High school	52 (30.4)	44 (23.0)	2.52	0.122
	≥College/University	119 (69.6)	147 (77.0)		
Employment status	Retired	152 (88.9)	181 (94.8)	4.22	0.052
	Other	19 (11.1)	10 (5.2)		
Family income	Low	124 (72.5)	140 (73.3)	0.03	0.906
	Moderate/High	47 (27.5)	51 (26.7)		
Resident status	Citizen/Permanent resident	160 (93.6)	187 (97.9)	4.28	0.061
	Other	11 (6.4)	4 (2.1)		
Birthplace	Mainland China	160 (93.6)	184 (96.3)	1.46	0.238
	Other	11 (6.4)	7 (3.7)		
Length in Canada	0–5 yrs	34 (19.9)	30 (15.7)	2.37	0.305
	6–15 yrs	88 (51.5)	94 (49.2)		
	>15 yrs	48 (28.1)	67 (35.1)		
Housing type	Apartment	75 (43.9)	61 (31.9)	24.43	<0.001
	House	87 (50.9)	85 (44.5)		
	Other	9 (5.3)	45 (23.6)		
Housing Size	1 person	24 (14.0)	28 (14.7)	1.43	0.490
	2 persons	86 (50.3)	106 (55.5)		
	3 persons or more	61 (35.7)	57 (29.8)		
Religion	No	117 (68.4)	140 (73.3)	1.04	0.354
	YES/Other	54 (31.6)	51 (26.7)		

**Table 2:** Wave Differences in Continuous Predictive Variables and Their Correlations with the Outcome Variables.

Continuous Variables	Wave Difference			Correlation *r*	
	Wave 1: 2020 Mean (SD)	Wave 2: 2023 Mean (SD)	*t*	WHO-5	SWLS
Age	74.23 (5.69)	75.19 (5.98)	−1.51	0.06	0.09
MSPSS	5.50 (0.68)	4.99 (1.04)	5.51 ***	0.52 ***	0.51 ***
MSPSS (Other)	5.52 (0.82)	4.92 (1.17)	5.55 ***	0.43 ***	0.44 ***
MSPSS (family)	5.70 (0.74)	5.31 (1.07)	3.95 ***	0.43 ***	0.42 ***
MSPSS (friends)	5.29 (0.80)	4.73 (1.20)	5.17 ***	0.53 ***	0.51 ***
WHO-5	17.62 (4.79)	13.13 (5.68)	8.05 ***		
SWLS	26.19 (4.57)	21.64 (5.12)	8.85 ***		

Note. *** *p* < 0.001.

## Data Availability

The data and data analysis files can be retrieved from https://osf.io/2ux5b/?view_only=bb0e29770ecc4befb62424d1583d8b36 (accessed on 11 March 2024). The full set of data collected in Wave 1 (2020) has been published before [9]. The data collected in Wave 2 (2022) was presented at the 52nd Annual Scientific and Educational Meeting of the Canadian Association on Gerontology. The merged data across the two Waves was submitted as a presentation to the 53rd Annual Scientific and Educational Meeting of the Canadian Association on Gerontology in 2024.
